# Experimental Study and Application of TPO Waterproofing Membrane Lapping Process Parameters

**DOI:** 10.3390/ma18143313

**Published:** 2025-07-14

**Authors:** Keyong Wang, Zhenhua Zang, Jie Li, Zhenyue Shi, Mingcai Liu, Zhipeng Li, Qingbiao Wang, Yandong Shang, Chenglin Tian, Zifan Jia, Hui Wang

**Affiliations:** 1 School of Resources, Shandong University of Science and Technology, Tai’an 271000, China; skd996607@sdust.edu.cn (K.W.); 202383300028@sdust.edu.cn (Z.Z.); 202283300056@sdust.edu.cn (J.L.); cornor@163.com (Y.S.); skdtcl@126.com (C.T.); 202483300045@sdust.edu.cn (Z.J.); 202383300027@sdust.edu.cn (H.W.); 2 Laboratory of Rock Burst Prevention Technology and Equipment, Shandong Energy Group Co., Ltd., Jinan 250200, China; 3 China Railway Construction Investment (Shandong) Dongyang Expressway Co., Ltd., Liaocheng 252299, China; 4 Jinan Campus, Shandong Jianzhu University, Jinan 250101, China; skd990748@sdust.edu.cn

**Keywords:** TPO waterproofing membrane, construction technology, peel strength, permeable path, impermeability test

## Abstract

Taking the TPO waterproofing membrane as an example, this paper studies the influence of temperature, speed and welding pressure on the welding quality of a TPO waterproofing membrane lap area through a peel test and a water impermeability test, determines the optimal construction process, and observes and compares the permeable path through laser confocal microscope. Finally, it is applied to the actual effect test in the project. The results show that the welding pressure test tool for the lap area of the waterproofing membrane is designed to meet the welding work test requirements of various lap areas of the waterproofing membrane. The peel strength increases first and then decreases with the increase in welding temperature, and the optimal construction temperature is 400 °C. The optimal construction speed is 4 m/min; at 400 °C welding temperature, the peel strength increases first and then decreases slightly with the increase in welding pressure. The optimal construction pressure is 14.97 N; under the condition of 0.2 MPa, 30 min to 0.6 MPa, 120 min, the water impermeability test of the overlapping area was qualified. In this paper, the optimal construction technology of a TPO waterproofing membrane is determined, which provides guidance for its application and promotion in engineering.

## 1. Introduction

Underground engineering waterproofing is an important process of engineering construction, and its construction quality is directly related to the safe operation of underground engineering in the future. A waterproofing membrane is one of the main means of waterproofing in underground engineering. Different types of membranes determine different construction techniques. At present, commonly used waterproofing membranes include modified asphalt waterproofing membranes, ethylene propylene diene monomer (EPDM) rubber waterproofing membranes, polyvinyl chloride (PVC) waterproofing membranes, etc., but there are many problems such as poor stability, complex processes, easy aging and high pollution [1,2,3].

The thermoplastic polyolefin (TPO) waterproofing membrane, as a new type of polymer waterproofing material, has been widely used in engineering in recent years due to its good aging resistance, excellent tensile strength, and environmental friendliness [1]. The waterproof performance of TPO waterproofing membrane depends largely on the welding quality of its lap joint, so a good welding process is the key to ensuring its engineering performance. However, at present, there is still a lack of systematic and standardized construction technology guidance for the hot-melt welding of TPO waterproofing membranes. In practical projects, problems such as missing welding, virtual welding, and over-welding often occur, which seriously affect the safety of structural waterproofing.

Previous studies have focused on the influence of a single welding parameter (such as temperature, speed or pressure) on the welding quality [4,5,6]. However, there is still a lack of systematic research on the coupling mechanism of the lap performance of TPO coils under the combination of multiple welding process parameters (such as temperature, speed and pressure). In this study, through the peel strength test and water permeability analysis, combined with laser confocal microscopy, the performance variation law of the TPO waterproofing membrane lap area under different temperature and speed combinations was revealed, aiming to provide theoretical basis and experimental basis for the formulation of standardized construction technology of the TPO waterproofing membrane, which has important engineering application value and practical significance.

In the construction technology of waterproofing membranes, the predecessors have carried out the following research: Michal et al. studied the influence of welding speed, welding temperature, welding time and other factors on the welding quality of waterproofing membranes through experiments and determined the most suitable combination of factors [7]. Zhang proposed a new leakage location online monitoring system, which integrates the detection core on the traditional waterproofing membrane to realize the function of leakage location monitoring [8]. Jiang studied the influence of a new polymer waterproofing membrane on the mechanical properties of tunnel lining structure [9]. Lee determined the applicability and contact conditions of waterproofing membranes under various conditions through numerical analysis [10]. Miyauchi proposed a method to determine the location of fasteners in waterproofing membranes [11]. Hailesilassie studied the influence of displacement rate and temperature of three different types of modified asphalt membranes on the bonding fracture energy by peeling test [12]. Tsukagoshi studied the carbon dioxide permeability of waterproofing membranes and carried out numerical simulation [13]. In the test method, according to the static mechanical test, Hopkinson impact test, and fly plate test used by Li W, the following test method is determined [14]: In terms of research and analysis, the force chain network analysis method is adopted [15]. Peng et al. carried out nanoindentation tests to evaluate the micromechanical properties of CASS and used the Split Hopkinson Pressure Bar (SHPB) device to study its dynamic mechanical behavior [16]. Li et al. studied the influence of karst caves in front of and along the tunnel on the stability of the surrounding rock by using the self-developed three-dimensional fluid–solid coupling model test system [17]. The changes in stress and seepage pressure of rock mass during tunnel excavation are analyzed. The synergistic effect of bentonite particle size and content, carboxymethyl starch (CMS) and Na_2_CO_3_ content on the properties of bentonite slurry was studied by rheological measurement and filtration test. The rheological behavior, parameters and the filtration loss of bentonite slurry with different factors were obtained. The range analysis was used to evaluate the influence of various factors on the slurry performance, and the best bentonite-based slurry ratio was proposed [18].

The number and content of the above research on the construction technology of the lap area of waterproofing membranes are relatively small, and the research mainly focuses on the waterproofing material and impermeability of waterproofing membranes. He et al. studied the effects of OMMT on the physical properties, chemical structure and microstructure of films before and after thermal aging, and they predicted the service life of SMB films based on the Arrhenius model [19]. Wang et al. focused on the coupling aging characteristics of SBS-modified asphalt waterproofing membranes, aiming to reveal its influence mechanism on material properties under high temperature and a freeze–thaw environment [20]. However, for the waterproofing membrane, the impermeability of the waterproofing membrane plays an extremely important role. There are few studies on the construction technology of the overlapping parts of the TPO membrane and the quality effect after construction. Only Garber used statistical methods to analyze the factors that promote and hinder the defects of the lap welding of the membrane during the production of cold-rolled strip steel in the bell-type furnace annealing [21]. Yang introduced in detail the connection problems, defects and countermeasures of synthetic polymer pre-laid waterproofing membranes in construction, which provided reference for the construction application and quality acceptance of synthetic polymer pre-laid waterproofing membranes [22]. Liu et al. constructed an effective deformation calculation model for the lap area of polyethylene polypropylene waterproofing membranes, considering the combined effects of lap size, process and environmental oxidation erosion [23]. In particular, the research on the construction temperature, speed and pressure parameters at the overlap of TPO waterproofing membrane still needs to be improved [24,25].

In the current engineering practice, the lap construction quality of TPO waterproofing membranes is directly related to the performance stability of the overall waterproofing system. In view of the influence of key parameters such as temperature, speed and pressure on the lap quality during the construction process, this paper systematically studies the optimal process parameters of TPO waterproofing membrane lap construction and verifies them through peel tests, the observation of permeable paths, and impermeability tests. The research results provide effective technical support for optimizing the lapping process of TPO waterproofing membranes, and they have important engineering application value for improving the construction quality of membranes and ensuring the durability of this project.

## 2. Materials and Methods

**Figure 1:**
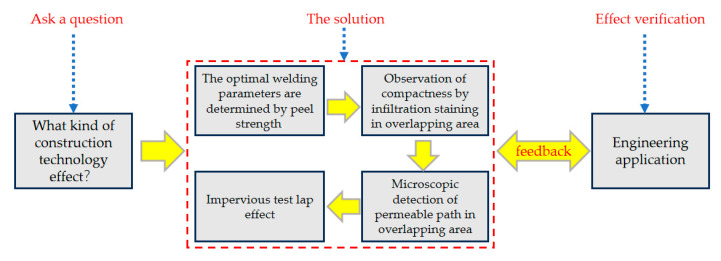
Technology roadmap.

In this study, the optimal welding temperature, speed and compression tests were carried out, and, combined with the penetration dyeing, permeable path detection and impermeability test of the lap area, the effects of different construction technologies on the impermeability of the lap area of waterproofing membranes were systematically evaluated. The aim is to determine the optimal construction technological parameters and provide technical support for subsequent related projects. Firstly, the optimal welding temperature and speed parameters of the lap area of the waterproofing membrane were determined by welding temperature and speed tests. Subsequently, based on the above two sets of optimal parameters, the welding pressure test was carried out to determine the optimal welding pressure. Secondly, according to the determined optimal construction technology, the impermeability of the lap area of the waterproofing membrane was tested under water immersion and thermal aging conditions to verify its waterproofing effect. Finally, the optimal construction process was derived based on the test results. The technical roadmap is shown in Figure 1.

### 2.1. Sample Specimen Preparation

#### 2.1.1. Sample-Making Equipment and Improvement

The preparation of TPO waterproofing membrane sample adopts the method of heating metal wedge with heating rod. By fully contacting the metal wedge with the polymer membrane, the surface of the membrane was melted and pressed, so as to promote the fusion of the overlapping surface of two adjacent membranes. Subsequently, under the action of the pressure roller, the welding surface was gradually compacted.

**Figure 2:**
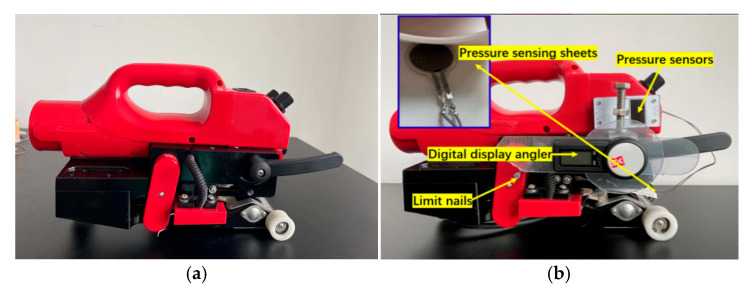
Sample-making equipment diagram: (**a**) Before the improvement of sample-making equipment. (**b**) After the improvement of sample-making equipment.

In order to determine the optimal welding process parameters, a test device was designed and built as shown in Figure 2. The device is composed of a climbing welding machine, an angle display, a limit device and a limit nail. Among them, the climbing welding machine equipment was provided by Wenzhou Mingqi Welding Equipment Co., Ltd., Wenzhou, China. Angle display, limit device and limit nail are produced by Delixi Electric Co., Ltd., Leqing City, Zhejiang Province, China. The temperature adjustment range is 150 °C–600 °C, and the welding speed range is 0.5 m/min–5 m/min; the digital angler was fixed to the pressure wrench for real-time monitoring of the welding angle. The pressure display was installed on the side wall of the welding machine to measure the welding pressure, as shown in Figure 2.

**Figure 3:**
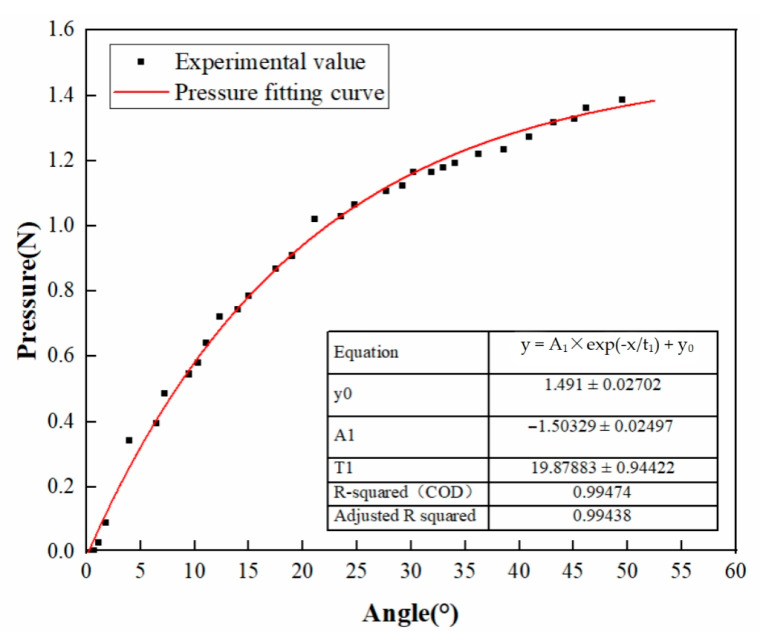
Fitting curve of the angle–pressure relationship for the climbing welding equipment.

During the test, the pressure sensor was placed between the upper and lower waterproofing membranes, and the pressure was accurately controlled by adjusting the rotation angle of the pressure wrench. The wrench angle when the pressure sensor first displays the pressure value was taken as the initial angle (set to 0°), and then the angle–pressure relationship curve is drawn (Figure 3).

In order to more accurately evaluate the relationship between the angle and pressure of the climbing welding equipment, the experimental data were fitted by an exponential function. The error range of each fitting parameter is shown in Figure 3. Specifically, the initial pressure term y_0_, the fitting coefficient A_1_ and the angle response coefficient t_1_ are 1.491 ± 0.02702, −1.50329 ± 0.02497 and 19.87883 ± 0.94422, respectively.

**Figure 4:**
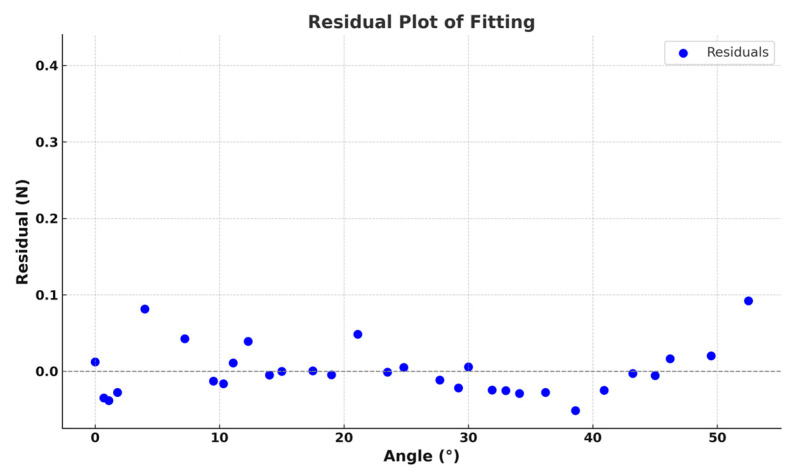
The fitting function residual diagram of the relationship between angle and pressure.

In addition to providing the fitting coefficients, this study also conducted a residual analysis, as shown in Figure 4. The residual standard deviation (SD) between the experimental and fitted values was calculated to quantify the fitting error, yielding a residual SD of 0.03752 N, with a maximum absolute residual error of 0.0685 N. The small residual errors indicate high fitting accuracy.

To reflect data uncertainty more clearly, error bars representing the standard deviation of repeated measurements were added to the graph. Each experimental data point was measured at least three times, and the error bars correspond to the calculated standard deviations. The error analysis followed the principles of the Guide to the Expression of Uncertainty in Measurement (GUM), incorporating both Type A (statistical) and Type B (instrumental) uncertainties.

The coefficient of determination R^2^ is 0.99474, and the adjusted R^2^ is 0.99438, confirming the reliability of the fitting model.

#### 2.1.2. Specimen Preparation

**Figure 5:**
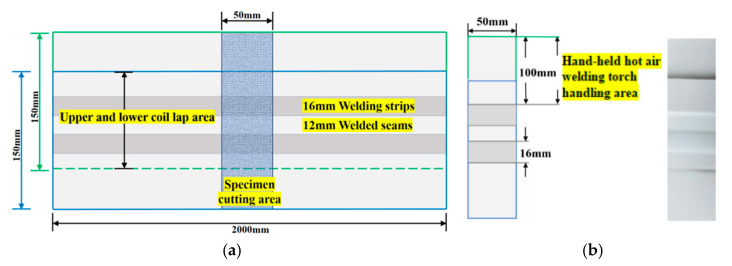
TPO waterproofing membrane welding specimen: (**a**) waterproofing membrane samples; (**b**) waterproofing membrane cutting test piece.

The test material used in this study is TPO-15H1 thermoplastic polyolefin waterproofing membrane (thickness 1.5 mm) produced by China Jiangsu Suzhou Karen Building Materials Co., Ltd. (Suzhou, China). The initial size of the waterproofing membrane is 150 mm × 2000 mm. According to the requirements of different test parameters, two waterproofing membranes were welded by the improved welding machine. After welding, two strip samples of 16 mm welding band and 12 mm detection seam were formed, as shown in Figure 5.

A rectangular specimen with a width of 50 mm was cut in the middle of each sample to ensure that the edge of the specimen reached 100 mm. The short side was welded by a hand-held hot air welding gun to meet the predetermined length requirements [26].

1. The optimal welding temperature and speed test sample preparation

When the welding temperature is 200 °C and the welding speed is 0.5 m/min, the heat accumulation in the welding area of the waterproofing membrane is insufficient, and the effective melting state cannot be achieved. Therefore, the minimum welding temperature is set to 300 °C, and the minimum welding speed is set to 1 m/min.

Under the condition of welding temperature of 600 °C and welding speed of 6 m/min, the surface of the waterproofing membrane was unevenly heated, and the bonding effect was poor due to the fast running speed of the equipment. And the welding temperature exceeds 600 °C, which is not conducive to on-site construction operation. Based on the above factors, the maximum welding temperature was set to 600 °C, and the maximum welding speed was set to 5 m/min. The preparation of the samples is detailed in Table 1.

2. Sample preparation of optimal welding pressure test

Based on the above test results, five types of waterproofing membrane lap specimens were prepared under the conditions of the determined optimal welding temperature and speed. By adjusting the rotation angle of the pressure handle to 10°, 20°, 30°, 40° and 50°, the welding pressure was controlled, respectively. The relationship curve between the rotation angle and the pressure is shown in Figure 3, and the preparation of the sample is detailed in Table 2.

3. Impermeability test sample preparation

According to the above test results, the optimal construction process of the lap area of the waterproofing membrane should meet the optimal welding temperature, speed and pressure parameters at the same time. The lap samples prepared under these process conditions were first aged in the thermal aging test chamber according to the established procedure, and then the impermeability test was carried out on the lap area after aging. The specific preparation and aging conditions of the samples are detailed in Table 3.

### 2.2. Test Scheme and Equipment

#### 2.2.1. Peel Test

**Figure 6:**
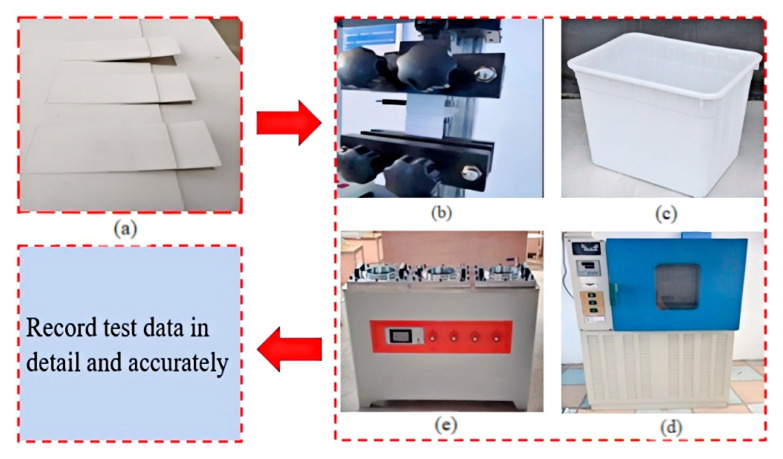
Test equipment diagram: (**a**) Specimen preparation uses TPO-15H1 thermoplastic polyolefin waterproofing membrane produced by China’s Jiangsu Province Suzhou Karen Building Materials Co., Ltd., Suzhou, China (**b**) Peeling test used WDW-05 microcomputer control universal testing machine produced by Jinan Wenteng Test Instrument Co., Ltd., Jinan, China. (**c**) The immersion test was carried out in an ordinary plastic box. (**d**) The thermal aging test adopts the high temperature aging test chamber produced by Rongjida Instrument Technology Co., Ltd., Minhang District, Shanghai City, China. (**e**) Impermeability test using China’s Shanghai Minhang District Rongjida Instrument Technology Co., Ltd., Shanghai, China, produced DFTS-1 waterproof membrane lap area impermeability instrument.

**Figure 7:**
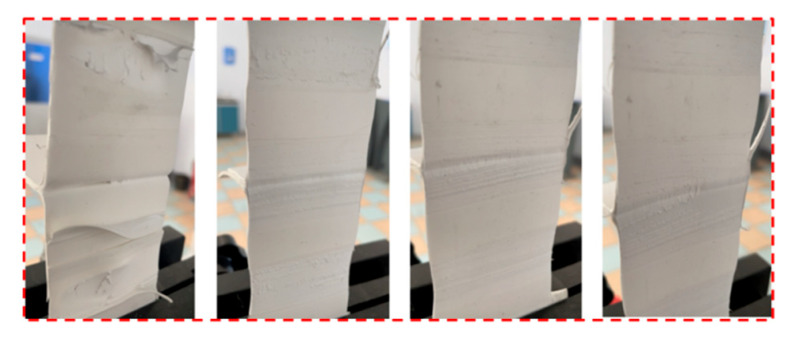
Partial peel test effect diagram.

The peel test adopts the WDW-05 microcomputer control universal testing machine, the instrument is produced in Jinan Wenteng Test Instrument Co., Ltd., Jinan, China. The maximum test force is 5000 N, and the accuracy of the force value is better than ±0.5% of the indicated value (see Figure 6b). Five rectangular specimens with a width of 50 mm perpendicular to the lap edge were cut from the middle of each lap specimen. The specimen was fastened to the fixture of the tensile testing machine to ensure that the longitudinal axis of the specimen coincides with the axis of the testing machine and the fixture. The distance between the fixtures is (100 ± 5) mm, and there is no preload. The test was carried out at a constant tensile speed of (100 ± 10) mm/min until the specimen was separated, and the failure modes of the lap area were recorded and analyzed, as shown in Figure 7.

**Figure 8:**
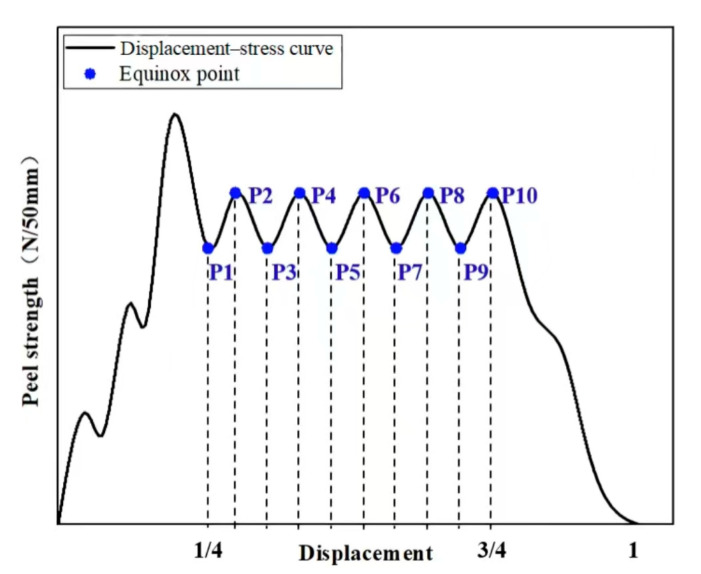
A schematic diagram for calculating the average peel strength.

The regions of [0–1/4) and (3/4–1] were removed, and the values at 10 equipartition points were retained as the average peeling strength. The average peel strength of 5 specimens in each group was taken as the final result, unit N/50 mm [27], as shown in Figure 8.

#### 2.2.2. Impermeability Test

According to the general specification for waterproofing of building and municipal engineering GB55030-2022 [27], the impermeability test was carried out by using DFTS-1 waterproof membrane lap area impermeable instrument, as shown in Figure 6e. Under the condition of 0.2 Mpa, 30 min, impermeability is qualified. There are two conditions:

1. Soaking treatment

The waterproofing membrane samples after welding and edge sealing were immersed in water at 23 °C, the water surface was 20 mm higher than the sample, and the samples were continuously immersed for 7 days. After removing, the surface moisture was sucked dry to ensure that there was no water stain on the surface of the sample. The test piece was cut with the middle of the lap as the center line, and then the impermeability test was carried out according to the specification requirements, as shown in Figure 6c.

2. Thermal aging treatment

The welded waterproofing membrane samples were placed in the aging box of model 401B (Jiangdu Jingang Machinery Factory, Nantong, China), and the condition was set at 80 °C for 7 days. After the aging, the impermeability of the sample lap was tested according to the requirements of the specification, as shown in Figure 6d.

## 3. Results and Discussion

According to the design scheme of the experimental group in Table 1, the peel strength test and impermeability test were carried out on the samples of the lap area of TPO waterproofing membrane. Through systematic processing and analysis of the collected test data, the performance differences in each experimental group under different test conditions are clarified. The specific test results and their analysis will be described in detail below.

### 3.1. Analysis of Peel Strength Test Results

#### 3.1.1. Variable Temperature and Variable Speed Peeling Test

**Figure 9:**
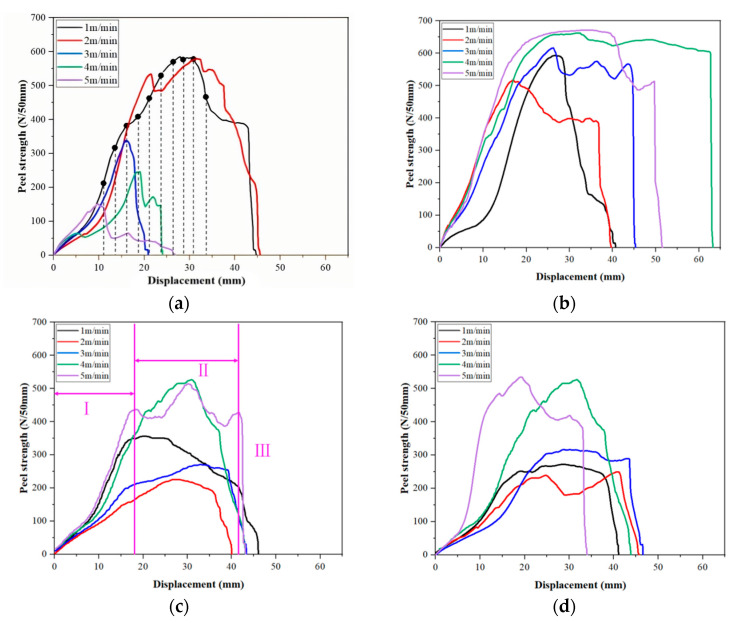
Displacement–stress curves at different temperatures and different speeds: (**a**) Peeling strength at different speeds at 300 °C. (**b**) Peeling strength at different speeds at 400 °C. (**c**) Peeling strength at different speeds at 500 °C, Region I is the initial stage of TPO waterproofing membrane failure. Region II is the stable stripping stage of TPO waterproofing membrane failure. Region III is the failure stage of TPO waterproofing membrane failure. (**d**) Peeling strength at different speeds at 600 °C.

Through the peel test of TPO waterproofing membrane specimens under different temperature and speed conditions, the displacement–stress data are obtained. Because the sample production equipment adopts double welding strips, and the peeling strength of the two welding strips is the same, only the test data of one of the welding strips are recorded during the test. The peeling test results of TPO waterproofing membrane specimens under various temperature and speed conditions are shown in Figure 9.

Taking the stripping curve at 500 °C and 5 m/min in Figure 9c as an example, the stripping process of the waterproofing membrane can be divided into three typical stages:

Region I (initial stage): This stage is the establishment process of the initial adhesion force, and the peel strength gradually increases with the displacement, indicating that the stress concentration occurs in the bonding layer during the loading process and gradually increases.

Region II (stable stripping stage): This stage presents a longer platform area, showing that the waterproofing membrane has better ductility and adhesion under these temperature and speed conditions. The existence of the platform area indicates that the waterproof membrane has undergone sufficient plastic deformation during the stretching process, which effectively avoids the brittle fracture phenomenon.

Region III (failure stage): As the displacement further increases, the peel strength decreases rapidly, indicating that the adhesive layer fails or completely peels off under continuous stretching.

Under the condition of 300 °C, the average peeling strength was 450.45 N/50 mm and 449.92 N/50 mm when the bonding speed was 1 m/min and 2 m/min, respectively. With the increase in bonding speed, the peeling strength is significantly improved, the peak position is obviously advanced, and the displacement is reduced. The higher bonding speed makes the peel strength curve show a steep trend, indicating that the waterproofing membrane has not yet completely softened, resulting in damage under a smaller displacement, which is manifested as the peak value of peel strength appearing in advance, reflecting the poor adhesion at this temperature.

Under the condition of 400 °C, when the bonding speed is 4 m/min and 5 m/min, the peeling strength is higher, and the average peeling strength is 628.08 N/50 mm and 607.13 N/50 mm, respectively. In the speed range of 1–2 m/min, the peeling strength reaches the peak, showing significant plastic deformation characteristics, and the peeling strength is up to more than 600 N/50 mm. Especially at the speed of 4–5 m/min, the peeling strength is further improved with the increase in speed. However, when the bonding speed reaches 5 m/min, the overall peeling strength decreases slightly due to the accelerated peeling process.

Under the condition of 500 °C, the average peeling strength at the bonding speed of 4 m/min and 5 m/min is 369.68 N/50 mm and 409.53 N/50 mm, respectively. Due to the slow bonding speed, the internal molecular group of the material is quickly broken, resulting in a low peak value of the peel strength; with the increase in bonding speed, the effect of high temperature on the material molecular group in a short time is relatively small, so the peeling strength shows an upward trend. However, the ductility of the material at this temperature is reduced, which can easily lead to early failure.

Under the condition of 600 °C, the average peeling strength at the bonding speed of 4 m/min and 5 m/min is 378.44 N/50 mm and 432.45 N/50 mm, respectively. At this temperature, the peel strength of the waterproofing membrane is similar to that at 500 °C, but the ductility of the material is slightly improved.

In summary, the peel strength at 400 °C is significantly higher than that at other temperature conditions, showing good adhesion and ductility.

**Figure 10:**
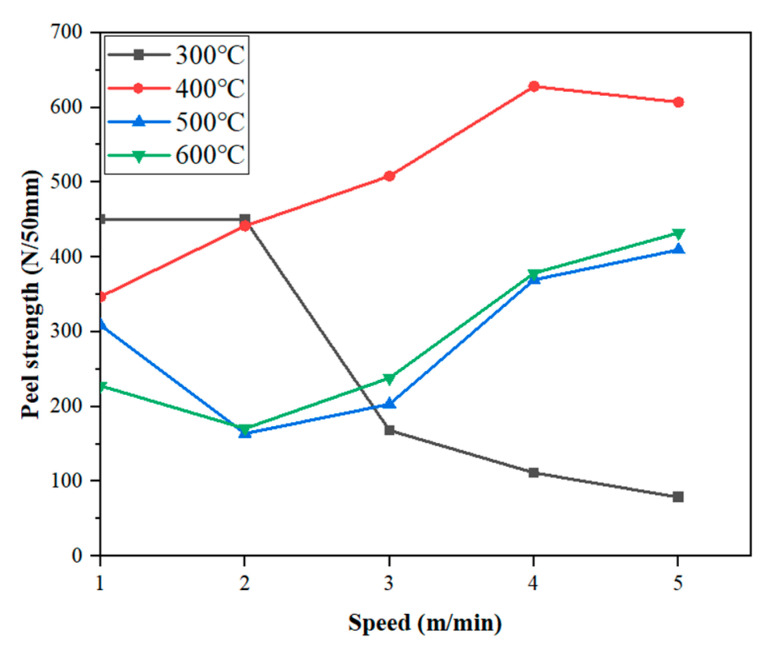
The velocity and peel strength diagram at each temperature.

From Figure 10, it can be seen that, at 300 °C, the peeling strength decreases with the increase in welding speed. This is because, at a constant temperature, the faster the welding speed of the equipment, the lower the heat energy absorbed by the waterproofing membrane in unit time. When the heat energy is lower than the melting point of the membrane, the number of macromolecular chains that creep and diffuse between the welding surfaces is less, and the interface layer of cooling fusion is thinner, so the strength is low, and the peeling performance is poor.

Under the condition of 400 °C, with the increase in the speed of the climbing welder, the average peeling strength reaches the peak at 4 m/min and then decreases slightly. This phenomenon can be attributed to the gradual diffusion of polymer molecules under external force after the contact surface of the TPO waterproofing membrane absorbs heat energy under high temperature conditions. With the increase in heat input or the extension of heating time, when the interface of the waterproofing membrane reaches the melt viscous flow state, not only do the mutual diffusion and penetration between the polymer melts occur, but also the macromolecular chains intertwine and diffuse across the interface melt layer to form a new molecular chain entanglement network, thus significantly improving the thermal welding peel strength. Subsequently, as the temperature gradually decreases, when the interface temperature is lower than the viscous flow temperature, the movement of macromolecular chains is limited, rearrangement, orientation and crystallization occur, and finally a dense melt interface layer with a certain thickness and strength is formed.

At 500 °C, with the increase in the speed of the welding machine, the average peel strength showed a continuous upward trend. However, when the welding speed is 1 m/min, the average peel strength of the waterproofing membrane decreases to 309.08 N/50 mm, which is the lowest value under this temperature condition. The reason is that, at a lower welding speed, the heat energy absorbed by the waterproofing membrane in unit time is too high, which leads to the fracture of the polymer molecular chain or the precipitation of small molecules, and the overflow layer is formed at the welding gap, thus weakening the adhesion strength at the lap area.

Under the condition of 600 °C welding, the change trend of peel strength with welding speed is similar to that at 500 °C. It is noteworthy that, at 600 °C, the peel strength exceeds that observed at 500 °C. This may be due to enhanced polymer flow and chain diffusion under extremely high heat input, promoting stronger bonding. Alternatively, it may be influenced by local temperature distribution or pressure inconsistencies. Further investigation is required to confirm whether this is a systematic effect or a measurement deviation.

**Figure 11:**
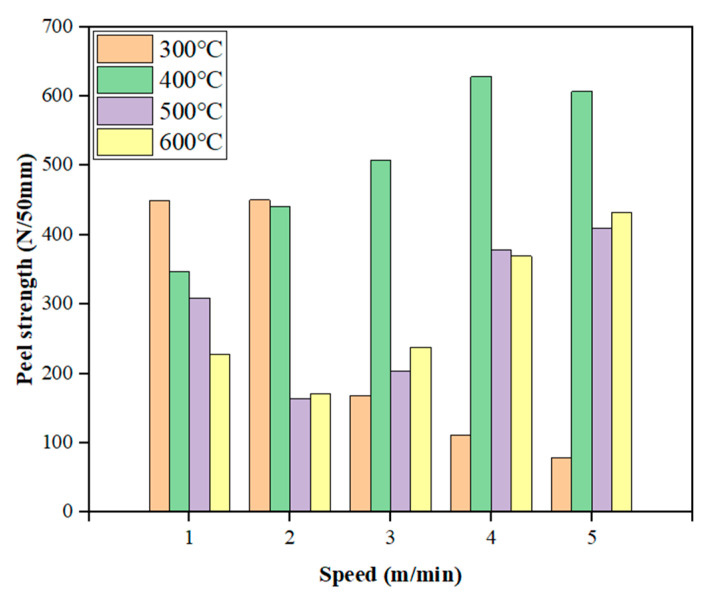
Temperature and peel strength diagram at each speed.

By each speed at different temperatures of TPO waterproofing membrane specimens, a peeling test was conducted to obtain its peel strength data. The peel test results of TPO waterproofing membrane specimens at different welding speeds and different temperatures are shown in Figure 11.

It can be seen from Figure 11 that, when the welding speed is 1 m/min, the waterproofing membrane with a welding temperature of 300 °C exhibits the highest average peel strength of 449.92 N/50 mm. Under the condition of the same speed, with the increase in welding temperature, the average peeling strength of the waterproofing membrane decreases in a stepwise manner. The reason is that, as the welding temperature increases, the heat energy absorbed by the welded surface of the waterproofing membrane increases, resulting in the fracture of the polymer molecular chain or the precipitation of small molecules, thereby reducing the peel strength.

Under the condition of a welding speed of 3 m/min, the highest value of average peeling strength appears in the waterproof membrane with a welding temperature of 400 °C, reaching 508.25 N/50 mm, showing obvious cliff-type improvement. This is mainly because the TPO waterproofing membrane, as a semi-crystalline polymer, must absorb heat energy higher than its melting point to form a molten layer that can undergo the peristaltic diffusion of macromolecular chains. If the heat energy is insufficient, an effective molten layer cannot be formed; on the contrary, if the heat energy is too high to exceed the cracking energy of the molecular chain, it will cause the molecular chain to break, and the complete melting layer cannot be formed. Therefore, the welding temperature plays a decisive role in the formation of the fusion layer at the welding interface, which in turn affects the quality and peel strength of the lap weld of the waterproofing membrane. The optimal welding temperature should ensure that the heat energy transferred to the waterproofing membrane is between its melting temperature and molecular chain-breaking temperature.

In addition, this law also shows a similar change trend under other welding speed conditions.

#### 3.1.2. Variable Pressure Stripping Test Under Better Conditions

**Figure 12:**
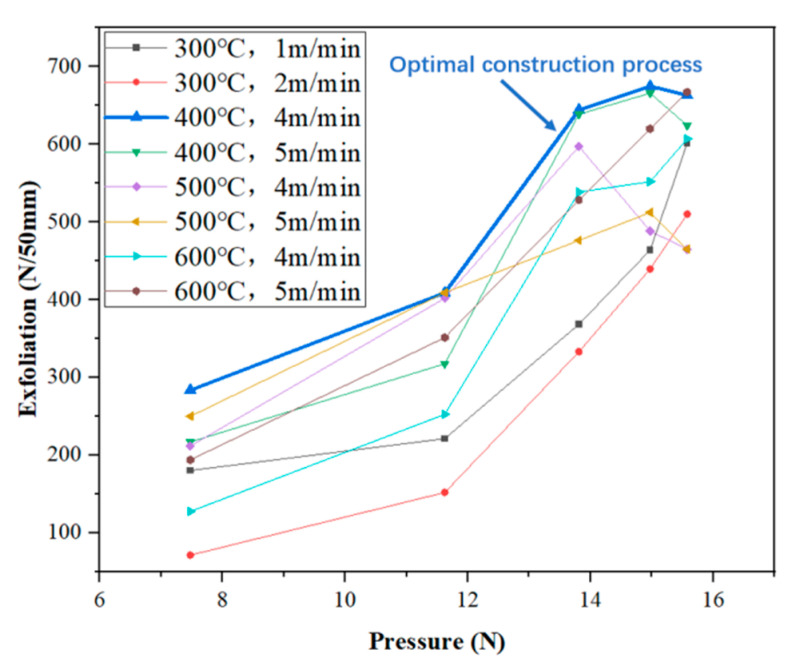
Different pressure-peel strength diagrams under better temperature and speed conditions.

Two groups of speeds with higher average peel strength in each group were selected at different temperatures to test the effect of different pressures on the peel strength. The results are shown in Figure 12.

It can be seen from Figure 12 that, when the welding temperature is 300 °C and the welding speed is 1 m/min and 2 m/min, the peel strength increases with the increase in welding pressure, but the peel strength when the welding speed is 1 m/min is always higher than that when the welding speed is 2 m/min.

When the welding temperature is 400 °C and the welding speed is 4 m/min, with the increase in welding pressure, the peeling strength continues to increase to the highest value and then decreases. The highest peeling strength is 674.92 N/50 mm, corresponding to the X-axis welding pressure of 14.97 N. The peeling process of TPO waterproofing membrane first passes through the elastic stage. After being pulled to the peak bearing capacity, the load is almost unchanged, the displacement increases, and it finally suddenly breaks after a period of platform period. Under the condition of a welding speed of 5 m/min, it also has a similar change rule, and the highest peeling strength is 666.06 N/50 mm.

When the welding temperature is 500 °C and the welding temperature is 4 m/min, with the increase in welding pressure, the maximum value of peeling strength is 597.49 N/50 mm when the welding pressure is 13.81 N. When the welding temperature is 5 m/min, the welding pressure reaches the maximum peeling strength of 512.53 N/50 mm at 14.97 N. The overall curve fluctuates greatly. In the early stage, the peeling force is small, and the peeling force rises rapidly. In the later stage, it gradually decreases until the two specimens are separated. The main reason for this result is that the temperature of the hot wedge welding method is difficult to control during construction. If the temperature is too high, the large molecular chain will decompose. If the temperature is too low, the surface of the waterproofing membrane will not melt completely, which is easy to cause hollowing and poor bonding, resulting in low peel strength.

When the welding temperature is 600 °C, whether the welding speed is 4 m/min or 5 m/min, the peel strength always increases with the increase in welding pressure and reaches the highest value when the welding pressure is 15.57 N.

### 3.2. Permeable Path Detection and Result Analysis

#### 3.2.1. Macroscopic Detection

The permeable dyeing observation of the lap area of the waterproofing membrane was carried out, and the nano-dyeing agent was dripped into the lap area of the waterproofing membrane to make it penetrate the lap area. The purpose is to evaluate the water permeability of the waterproofing membrane and the sealing effect of the lap area through the penetration test.

**Figure 13:**
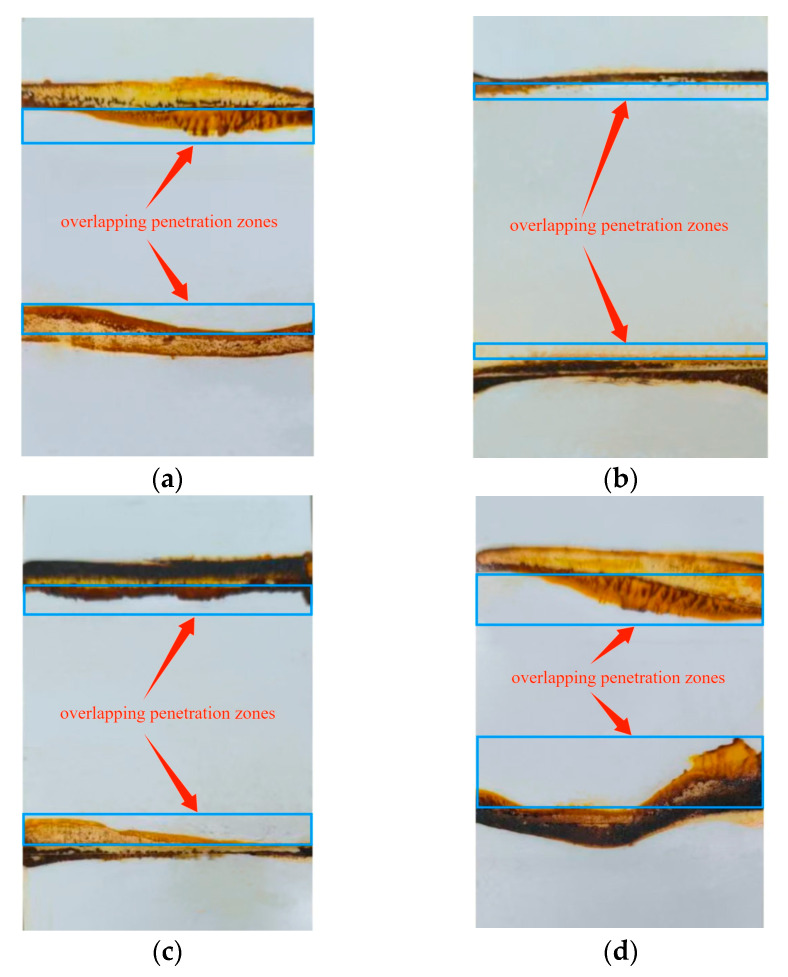
Permeability of lap area of waterproofing membrane: (**a**) 300 °C, 1 m/min; (**b**) 400 °C, 4 m/min; (**c**) 500 °C, 5 m/min; (**d**) 600 °C, 5 m/min.

According to Figure 9, the welding speed with the highest peel strength was selected as the test sample of penetration path detection at each temperature; that is, the waterproof membrane lapped at 300 °C, 1 m/min (300 °C, 1 m/min indicates a welding temperature of 300 °C, a welding speed of 1 m/min); 400 °C, 4 m/min; 500 °C, 5 m/min; 600 °C, 5 m/min, and the welding pressure is 14.97 N permeable path detection, as shown in Figure 13.

The larger the dyeing area of the lap penetration zone is, the worse the lap compactness of the waterproofing membrane is. The evaluation of the penetration range and sealing performance under each parameter group is shown in Table 4:

From the observation of the penetration results, especially at the edges of both sides, there is a significant dyeing diffusion phenomenon, in which the temperature is too high and the dyeing area is irregular and shows obvious bending, indicating that the penetration phenomenon of the lap area is the most serious.

#### 3.2.2. Microscopic Detection

**Figure 14:**
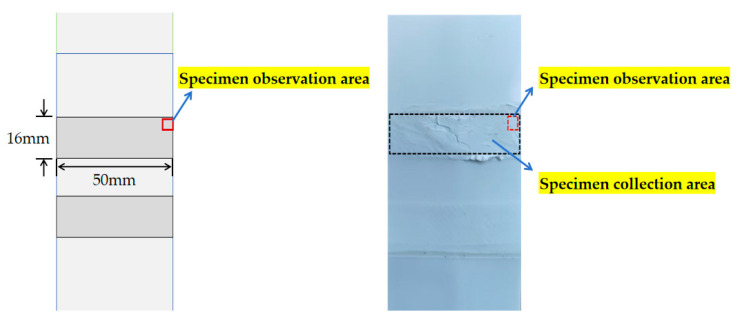
Microscopic detection of sample location.

The lap area sample (size: 50 mm × 16 mm × 1.5 mm) of the stripped waterproofing membrane is characterized by laser confocal microscopy (Japan Olympus OLS4100 produced in China Henan Zhengzhou e Test Co., Ltd., Zhengzhou, China) at the relatively weak water permeability of the lap edge, as shown in Figure 14.

**Figure 15:**
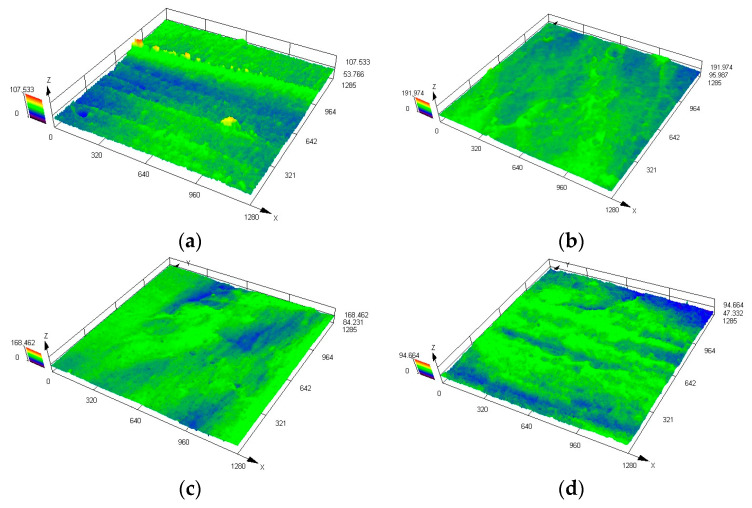
Three-dimensional topography of lap area of waterproofing membrane: (**a**) 300 °C, 1 m/min; (**b**) 400 °C, 4 m/min; (**c**) 500 °C, 5 m/min; (**d**) 600 °C, 5 m/min.

The purpose of this study is to reveal the microscopic permeable path of the waterproofing membrane and to provide a basis for the standardization of welding construction technology, so as to prolong the service life of waterproofing membranes. Figure 15 shows the three-dimensional morphology of the sample. The blue area corresponds to a lower relative height, indicating that the overlap density is insufficient, and the water forms a permeable path along the low-lying part. The X, Y and Z triaxial units in the figure are all μm, and the magnification is 200 μm.

It can be seen from the three-dimensional topography of Figure 15 that the surface morphology of the lap interface is significantly different under different welding temperature and speed combinations. The maximum height and morphological characteristics of the Z-axis under each parameter group are shown in the following Table 5:

#### 3.2.3. Analysis of Results

In the construction of a TPO waterproofing membrane lap area, the area with large penetration is concentrated on the edge of the waterproofing membrane, which is obviously closely related to the process parameters such as welding temperature, welding speed and welding pressure. Therefore, in order to improve the sealing and waterproofing performance of the waterproofing membrane, especially the sealing effect in the edge part, the optimized construction process must be strictly followed.

Through the comparison of Figure 15, it can be found that, with the appropriate increase in welding temperature and speed, the surface depression area of the lap area gradually decreases, and the blue low-lying area obviously shrinks, indicating that the number of permeable paths gradually decreases. Especially under the condition of 400 °C and 4 m/min, the lap area shows a relatively smooth three-dimensional morphology, the blue area is the least, and the overall height difference in the interface is small, indicating that the weld area under this process combination has good compactness and optimal welding quality. In contrast, the interface formed at 300 °C and 1 m/min has obvious ups and downs. This is due to insufficient welding heat input, resulting in insufficient melting of the material, forming a large number of unmelted areas and voids, which in turn causes more tiny water paths and reduces the sealing performance of the waterproofing membrane. Furthermore, under the condition of 600 °C and 5 m/min, although the permeable path is less and the Z-axis height fluctuation is the smallest, the excessive heat input may lead to material over-melting or thermal damage, so that the waterproofing membrane forms a longer flow path, which in turn affects the microstructure stability and long-term reliability of the waterproofing membrane.

The analysis results of the morphology characteristics show that, when the welding temperature is 400 °C and the welding speed is 4 m/min, the morphology of the lap area of the waterproofing membrane is the most ideal, the dyeing area is the smallest, the permeable path is the least, and the interface compactness is the highest, which can be used as the recommended welding parameters. In order to ensure the welding quality and avoid thermal stress concentration or insufficient heat input caused by too high or too low temperatures, it is recommended to avoid the use of a ‘high temperature + low speed‘ or ‘low temperature + high speed‘ combination process; otherwise, it will be difficult to guarantee the welding quality and waterproof performance of the lap area.

Based on the test results of the experimental group, the following optimal welding parameters are recommended:

Welding temperature: 400 °C, to ensure that the waterproof membrane fully melted and to improve the sealing performance; welding speed: 4 m/min, to avoid a too-fast welding speed, leading to uneven weld or a waterproof membrane not fully fused.

### 3.3. Analysis of Test Results of Impermeability Test

The waterproof membrane lap area based on the optimal construction technology was tested for impermeability after immersion treatment and thermal aging treatment. The test results are shown in Table 6. The results show that the overlapping area shows good impermeability under the standard requirements (0.2 MPa, 30 min) and higher requirements (0.6 MPa, 120 min), which fully verifies the effective improvement of the waterproof performance of the overlapping area of the waterproofing membrane by optimizing the construction process.

### 3.4. Engineering Application

#### 3.4.1. Project Profile

The project is located in Lingshanwan Road, Huangdao District, Qingdao City, Shandong Province, from Dazhushan Middle Road in the west to Dongyue East Road in the east. The utility tunnel plays an important role in urban construction. The pipelines of water, electricity, gas and heating are summarized in the utility tunnel structure, which solves the problem of complex pipelines in urban infrastructure construction. However, the underground environment of the utility tunnel is complex, especially near the coast, and the groundwater level is high, which seriously threatens the long-term safe operation of the utility tunnel.

**Figure 16:**
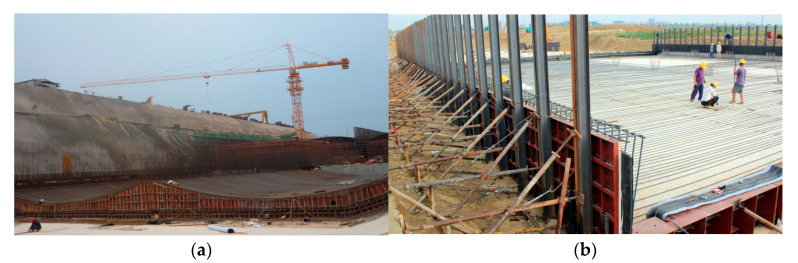
Construction site drawing: (**a**) Construction stage. (**b**) Completion stage.

Therefore, the waterproof engineering of the pipe gallery is an important step in its construction, and it must be constructed with high quality. In view of the requirements of the utility tunnel project for the impermeability of waterproof materials, the TPO waterproofing membrane was finally adopted after comprehensive consideration of the test results and design requirements. As shown in Figure 16, the hot wedge welding process with a welding temperature of 400 °C, welding speed of 4 m/min and welding pressure of 14.97 N was used for the construction of the overlapping area.

#### 3.4.2. Effect Inspection

The results obtained by the test are applied to the pipe gallery project, and both the construction efficiency and the construction quality of the workers have been greatly improved. The waterproof coil sampling test is carried out after the completion of the project construction. The test results are shown in Table 7, and they meet the requirements.

#### 3.4.3. Effect Contrast

In this section, the bonding properties of different waterproofing membranes were compared [28,29,30], and the specific results are shown in Table 8. The test included two indicators: peel strength and impermeability (no treatment). Obviously, the peel strength of the TPO waterproofing membrane welded under the optimal construction process is significantly higher than that of other waterproofing membranes.

## 4. Conclusions

The main conclusions obtained by testing the mechanical properties of the overlapping area of the TPO waterproofing membrane are as follows:

(1) The experimental results show that there is an obvious nonlinear relationship between the peel strength of the waterproofing membrane and the welding temperature: with the temperature from low to high, the peel strength increases first and then decreases, indicating that there is an optimal welding temperature. The results show that the best welding temperature is 400 °C. At this time, the welding quality of the waterproofing membrane is the best, and the peel strength reaches its peak. Under different temperature conditions, the sensitivity of peel strength to welding speed is also different: at 300 °C, the peel strength continues to decrease with the increase in speed; at 400 °C, the peeling strength first increases with the increase in speed to the peak and then decreases. At 500 °C and 600 °C, it shows a complex mode of decreasing first and then rising. Considering the welding quality and construction efficiency, it is recommended to use 400 °C and 4 m/min as the best welding parameters to fully guarantee the mechanical properties and waterproof performance of the welded lap. Considering the welding quality and construction efficiency, it is recommended to use 400 °C and 4 m/min as the best welding parameters to fully guarantee the mechanical properties and waterproof performance of the lap area.
(2) In this paper, the influence of welding pressure on the peel strength of the waterproofing membrane was studied, and the regulation effect of welding temperature was analyzed. The results show that the peel strength increases with the increase in welding pressure at 300 °C and 600 °C. At 400 °C and 500 °C, the peel strength increased first and then decreased slightly. The comprehensive analysis shows that the optimal construction pressure is 14.97 N, and the optimal peel strength can be obtained under this pressure, which provides an effective reference for optimizing the welding process of waterproofing membranes.
(3) The experimental results of penetration path detection show that the edge of the lap area is the area with the largest penetration. It is found that the combination of a ‘high temperature + low speed’ or ‘low temperature + high speed’ welding process can easily lead to penetration problems, which further emphasizes the importance of the strict implementation of welding standards in the actual construction process. Especially at the edge of the lap zone, the welding process control should be strengthened to effectively reduce the penetration risk, prolong the service life of the waterproofing membrane and improve its waterproofing performance.
(4) After the impermeability test, it is verified that there is no leakage in the welding lap of the waterproofing membrane under the working conditions of 0.2 MPa, 30 min to 0.6 MPa and 120 min. All the indexes meet the relevant qualification standards, indicating that the lap process can effectively guarantee the waterproofing performance and has good engineering application value.
(5) In this paper, a special pressure test tool for the welding performance test of the lap area of a waterproofing membrane is designed. The tool can be effectively applied to the welding performance test of various common waterproofing membrane materials including high-density polyethylene (HDPE), polyvinyl chloride (PVC) and ethylene propylene diene monomer (EPDM). By providing accurate welding pressure data, the test tool can provide a scientific basis and technical support for optimizing welding parameters, improving the welding quality and waterproofing performance of waterproofing membranes.

It can be directly applied to roofing, basement, tunnel and other projects involving TPO waterproofing membranes and guide the construction side to select the optimal welding temperature (400 °C), welding speed (4 m/min) and pressure (14.97 N) to ensure that the lap area has good anti-stripping and impervious performance. The research results can provide data support for the construction specifications and technical guidelines of TPO waterproofing membranes and other polymer waterproofing membranes, as well as help to promote the standardization and standardization of construction in the industry. The peeling strength test, impermeability test method and microscopic path observation method proposed in this paper can be used as one of the quality inspection methods of waterproof engineering, which can be used for on-site sampling and quality supervision. The designed welding pressure test tool and parameter response mechanism can be applied to the development of construction equipment to improve the adaptability and automation of equipment. In the future, multi-factor coupling numerical simulation and the optimization of temperature, velocity, pressure and interface quality can be carried out based on experimental data to improve the prediction ability of theoretical models.

## Figures and Tables

**Table 1:** Sample 1 preparation and test type table.

Sample Number	Welding Temperature (°C)	Welding Speed (m/min)	Quantity
Sample 1–300	300	1	5
		2	5
		3	5
		4	5
		5	5
Sample 1–400	400	1	5
		2	5
		3	5
		4	5
		5	5
Sample 1–500	500	1	5
		2	5
		3	5
		4	5
		5	5
Sample 1–600	600	1	5
		2	5
		3	5
		4	5
		5	5

**Table 2:** Specimen 2 preparation and test type table.

Sample Number	Pressure Handle Rotation Angle (°)	Corresponding Welding Pressure (N)	Quantity
2-1	10°	7.47	5
2-2	20°	11.62	5
2-3	30°	13.81	5
2-4	40°	14.97	5
2-5	50°	15.57	5

**Table 3:** Specimen 3 preparation and test type table.

Sample Number	Test Name	Test Environment and Conditions	Quantity
3-1	No treatment	Air, 23 °C, 7 d	5
3-2	Soaking treatment	Water, 23 °C, 7 d	5
3-3	Thermal aging treatment	Aging apparatus, 80 °C, 7 d	5

**Table 4:** Permeability summary and comparison.

Process Parameters	Permeability Regions	Sealing Performance Evaluation
300 °C, 1 m/min	Larger	Poor
400 °C, 4 m/min	Minimal	Good
500 °C, 5 m/min	Smaller	Better
600 °C, 5 m/min	Maximum	Very poor

**Table 5:** Summary and comparison of three-dimensional morphology characteristics.

Process Parameters	Maximum Height of Z-Axis (μm)	Surface Undulation Characteristics	Assessment of Permeable Path in Lap Area
300 °C, 1 m/min	107.53	Large ups and downs, local protrusions and depressions coexist	Multiple, there is obvious direct current penetration path.
400 °C, 4 m/min	191.97	The morphology is uniform, less concave and convex	Very little, the interface is closely integrated.
500 °C, 5 m/min	168.46	The flatness is high, but the local fluctuation is large	Fewer, the compactness is good, but there is a short flow path.
600 °C, 5 m/min	94.66	Fluctuations, uneven distribution	Moderate, there are local voids to form a flow around, and the path is long.

**Table 6:** Test results of impermeability of overlapping area of waterproofing membrane.

Construction Technology of Lapping Area	Test Conditions and Results					
Welding temperature 400 °C Welding speed 4 m/min Welding pressure 14.9 N	No treatment 0.2 Mpa 30 min	No treatment 0.6 Mpa 120 min	Soaking treatment 0.2 Mpa 30 min	Soaking treatment 0.6 Mpa 120 min	Thermal aging treatment 0.2 Mpa 30 min	Thermal aging treatment 0.6 Mpa 120 min
	Qualified	Qualified	Qualified	Qualified	Qualified	Qualified

**Table 7:** Test results of waterproofing membrane in construction site.

Construction Technology of Lapping Area	Peeling Test Results	Test Conditions and Results of Impermeability Test	
Welding temperature 400 °C Welding speed 4 m/min Welding pressure 14.9 N	671.37 N/50 mm	Test conditions: Soaking treatment 0.2 Mpa, 30 min	Test conditions: thermal aging treatment 0.2 Mpa, 30 min
		Qualified	Qualified

**Table 8:** Comparison of effects of different waterproofing membranes.

Type	Peel Strength	Impermeability (No Treatment)
TPO waterproofing membrane	671.37 N/50 mm	0.6 MPa, 120 min
SBS oil-soaked waterproofing membrane	180 N/50 mm	0.2 MPa, 30 min
Coating + SBS waterproofing membrane	90 N/50 mm	0.4 MPa, 30 min
POEA-PET	118 N/50 mm	0.3 MPa, 120 min

## Data Availability

The original contributions presented in this study are included in this article. Further inquiries can be directed to the corresponding author.

## Abbreviations

EPDM	Ethylene propylene diene monomer
PVC	Polyvinyl chloride
TPO	Thermoplastic polyolefin
SHPB	Split Hopkinson pressure bar
CMS	Carboxymethyl starch
OMMT	Organo-montmorillonite
SBS	Styrene–Butadiene–Styrene
SD	Standard deviation
POEA-PET	Palm oil-based polyurethane composite waterproofing membrane
